# *Radix Bupleuri*: A Review of Traditional Uses, Botany, Phytochemistry, Pharmacology, and Toxicology

**DOI:** 10.1155/2017/7597596

**Published:** 2017-05-16

**Authors:** Fude Yang, Xiaoxv Dong, Xingbin Yin, Wenping Wang, Longtai You, Jian Ni

**Affiliations:** ^1^Gansu University of Chinese Medicine, Lanzhou 730000, China; ^2^School of Chinese Materia Medica, Beijing University of Chinese Medicine, Beijing 100102, China

## Abstract

*Radix Bupleuri *(Chaihu) has been used as a traditional medicine for more than 2000 years in China, Japan, Korea, and other Asian countries. Phytochemical studies demonstrated that this plant contains essential oils, triterpenoid saponins, polyacetylenes, flavonoids, lignans, fatty acids, and sterols. Crude extracts and pure compounds isolated from* Radix Bupleuri *exhibited various biological activities, such as anti-inflammatory, anticancer, antipyretic, antimicrobial, antiviral, hepatoprotective, neuroprotective, and immunomodulatory effects. However,* Radix Bupleuri* could also lead to hepatotoxicity, particularly in high doses and with long-term use. Pharmacokinetic studies have demonstrated that the major bioactive compounds (saikosaponins a, b_2_, c, and d) were absorbed rapidly in rats after oral administration of the extract of* Radix Bupleuri*. This review aims to comprehensively summarize the traditional uses, botany, phytochemistry, pharmacology, toxicology, and pharmacokinetics of* Radix Bupleuri* reported to date with an emphasis on its biological properties and mechanisms of action.

## 1. Introduction

*Radix Bupleuri*, also called “Chaihu” in Chinese, is derived from the dried roots of* Bupleurum chinense* DC. and* Bupleurum scorzonerifolium *Willd. [1]. As a traditional herbal medicine,* Radix Bupleuri *has been used widely for the treatments of influenza, fever, inflammation, malaria, menstrual disorders, and hepatitis in China, Japan, Korea, and other Asian countries [2, 3]. According to ancient Chinese medical literatures,* Radix Bupleuri *is capable of regulating the exterior and interior metabolisms, dispersing evil heat from the superficies, soothing the liver, and promoting yang and qi (representing “life energy” or “life force” in TCM theories). In recent decades, investigations of* Radix Bupleuri *have focused on its biological activities, including its anti-inflammatory [4, 5], anticancer [6, 7], antipyretic [8], antimicrobial [9], antiviral [10], hepatoprotective [11], and immunomodulatory effects [12]. In addition,* Radix Bupleuri *also exhibited significant effects on membrane fluidity [13]. These studies have resulted in the isolation of essential oils, triterpenoid saponins, polyacetylenes, flavonoids, lignans, fatty acids, and sterols from this plant [14]. Among them, triterpenoid saponins are known to be the major bioactive compounds [15, 16]. Saikosaponins a and d are commonly used as chemical standards for quality evaluation of* Radix Bupleuri* in the current Chinese Pharmacopoeia and recent publications. However, an increasing number of recently published studies have reported adverse effects of* Radix Bupleuri*. The purpose of this review is to provide updated, comprehensive information on the traditional uses, botany, phytochemistry, pharmacology, toxicology, and pharmacokinetics of* Radix Bupleuri *based on scientific literatures in the past few decades. This study will facilitate exploring the therapeutic potential of this plant and evaluate future research opportunities.

## 2. Traditional Uses

*Radix Bupleuri*, which is characterized by a wide spectrum of biological and pharmacological effects, has been used as a famous traditional Chinese medicinal herb with a history of medical use in China. According to TCM theory,* Radix Bupleuri* is thought to regulate the exterior and interior metabolisms, disperse evil heat from superficies, sooth the liver, and promote yang and qi [1].* Radix Bupleuri* was initially recorded in Shennong Bencao Jing, a famous monograph of traditional Chinese medicine written in China in 200 AD [17]. According to a record of traditional Chinese medicine dated 2000 years ago,* Radix Bupleuri* had mainly been used for the treatment of mouth-bitterness, throat-drying, and eyes-dazzling. In other monographs of Materia Medica, such as Jin Kui Yao Lue, Kaibao Bencao, Bencao Gangmu, and Xinbian Bencao, this plant was described to be used for the treatment of common cold with fever, influenza, hepatitis, malaria, menoxenia, and hyperlipidemia [18]. Currently,* Radix Bupleuri* exerts other pharmacological effects, such as balancing different organs and energies within the body, strengthening the action of the digestive tract, improving liver and circulatory system function, and relieving liver tension [19]. Therefore, it is also used as a popular tonic herb in China. In Korea and Japan, this plant is commonly used for the treatment of fever, pain, and inflammation associated with influenza and the common cold [20]. In addition, this plant is also used as analgesics in the treatment of distending pain in the hypochondriac region of the chest and against amenorrhoea.

*Radix Bupleuri* is combined with other herbs in many classical formulas to improve clinical effect. Xiaoyaosan (XYS), a well-known formula for relieving depression, is originated from the book of “Taiping Huimin Heji Jufang” in Song Dynasty (960–1279 AD), consisting of* Radix Bupleuri*,* Radix Angelicae Sinensis*,* Radix Paeoniae Alba*,* Rhizoma Atractylodis Macrocephalae*,* Poria*,* Radix Glycyrrhizae*,* Herba Menthae*, and* Rhizoma Zingiberis Recens*. XYS exerts various pharmacological effects, including soothing the liver and improving the circulation of qi to relieve depression. Furthermore, it has been commonly recognized as a safe and effective prescription in the treatment of depressive disorder [21–23]. The 2015 edition of the Chinese Pharmacopoeia lists 97 Chinese patent medicines containing* Radix Bupleuri*, and the compatible herbs that are more frequently described include* Radix Glycyrrhizae*,* Rhizoma Zingiberis Recens*,* Radix Scutellariae*, and* Panax ginseng*.  Table 1 lists subsets of the Chinese patent drugs containing* Radix Bupleuri* in different formulations. Although* Radix Bupleuri* has been used to treat certain diseases in ethnomedicines for thousands of years, this herbal medicine should be comprehensively understood and recent information should be obtained for its clinical use.

## 3. Botanical Characterization and Distribution

**Figure 1:**
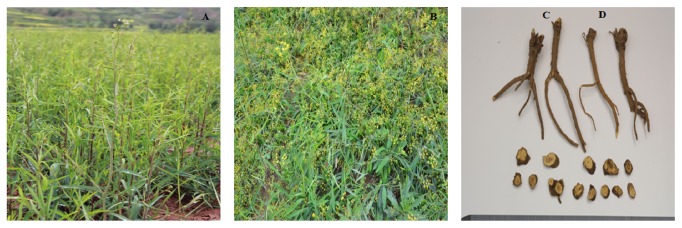
The whole plant of* Bupleurum chinense* DC. (A); the whole plant of* Bupleurum scorzonerifolium *Willd. (B); the roots of* Bupleurum chinense* DC (C); the roots of* Bupleurum scorzonerifolium *Willd. (D).

According to the Chinese pharmacopoeia,* Radix Bupleuri *is derived from the dried roots of* Bupleurum chinense* DC. and* Bupleurum scorzonerifolium *Willd. (Figure 1). The former is known as “Beichaihu” (Northern Chinese Thorowax Root) and the latter is known as “Nanchaihu” (Southern Chinese Thorowax Root), due to the difference of the origin and description. The drug is collected in spring or autumn, removed from the aerial part and soil, and dried.

*Beichaihu*. As a herbaceous perennial plant,* Bupleurum chinense* DC. grows to the height of 50–85 cm. Its root is conical, taupe, and approximately 6–20 cm in length. Its stems are erect, tufted, and apex branched. The leaves are alternate; the leaf blades are broadly linear-lanceolate, 4–7 × 0.6–0.8 cm in size; the apex is acuminate. Compound umbels are axillary and terminal, approximately 1–4 cm in length; the bracts are 0 or 2-3, linear; the petals are 5 and bright yellow. The fruits are oblong, brown, and approximately 3 mm in length. They blossom from July to September and fruit from August to October. This plant is widely cultivated in China, Japan, and Korea. Moreover, it grows in grasslands, stream banks, sunny slopes and roadsides, and other locations with altitudes of 100–2700 m [24].

*Nanchaihu*.* Bupleurum scorzonerifolium *Willd. is a herbaceous perennial plant and approximately 30–60 cm high. Its root is flexuose, reddish brown, and approximately 4–10 cm in length. Its stems are 1–3, usually glabrous, and apex branched. The leaf blades are linear or linear-lanceolate, 6–16 × 0.2–0.7 cm in size; the apex is acuminate. Compound umbels are axillary and terminal; the bracts are 1–3; the petals are 5 and yellow. The fruits are ellipsoid, dark brown, approximately 2-3 mm in length. They blossom from July to August and fruit from August to October. This plant is also widely cultivated in East Asian countries, such as China, Japan, and Korea. It grows in shrub forest margins, sunny mountain slopes, dry grasslands, and other locations with altitudes of 200–3000 m [24].

There were approximately 36* Bupleurum* species and varieties in different districts and markets due to the slightly different morphologic appearance of* Bupleurum* plants, including* B. longiradiatum *Turcz. with toxic ingredients and* B. hamiltonii* Balak with very little active constituents [25, 26]. Inevitably, this situation will compromise the values of* Radix Bupleuri *and even imperil the safety of the consumers. Until now, several techniques have been developed to identify and distinguish them, including TLC [27], HPLC [28–30], HPLC-ELSD [31, 32], HPLC-MS [33, 34], UPLC-MS [35], and capillary electrochromatography [36]. Among these methods mentioned above, HPLC-ELSD is the most commonly used analytical method for evaluating the quality and authenticity of* Radix Bupleuri*. The total amounts of saikosaponins a and d are used as the indicator compounds to characterize the quality of this plant with minimum contents of 0.3% in the Pharmacopoeia of People's Republic of China.

## 4. Chemical Constituents

**Figure 2:**
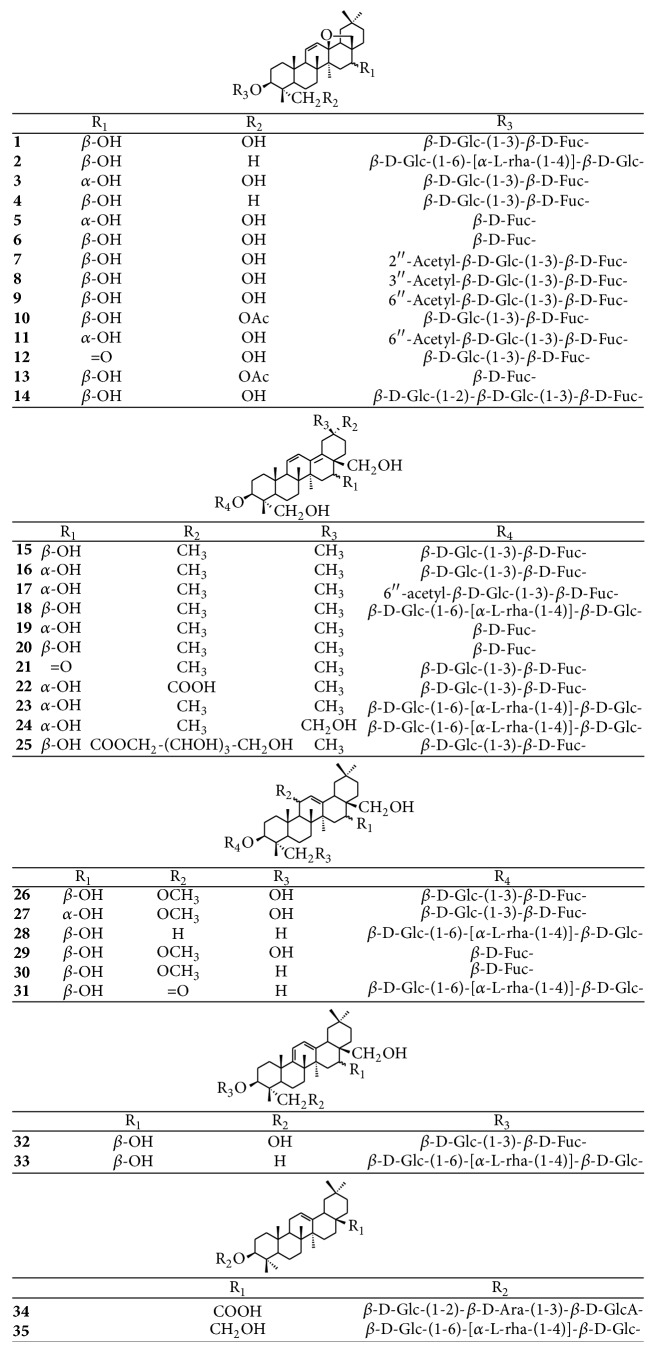
Chemical structures of triterpenoid saponins.

**Figure 3:**
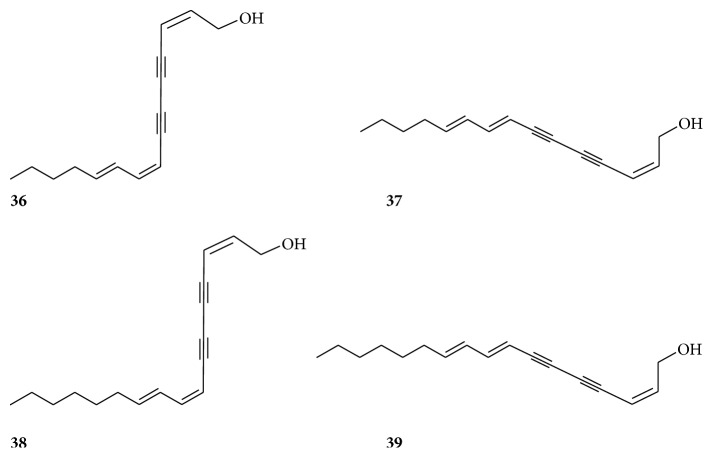
Chemical structures of polyacetylenes.

**Figure 4:**
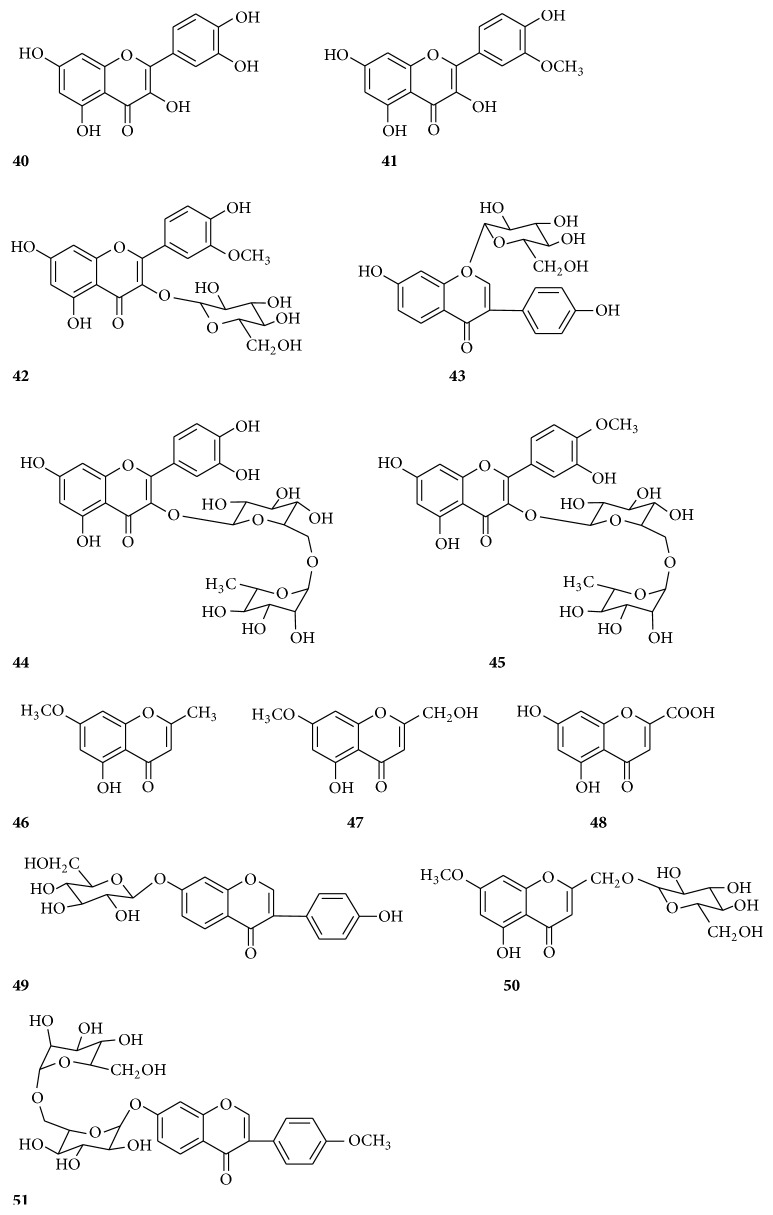
Chemical structures of flavonoids.

**Figure 5:**
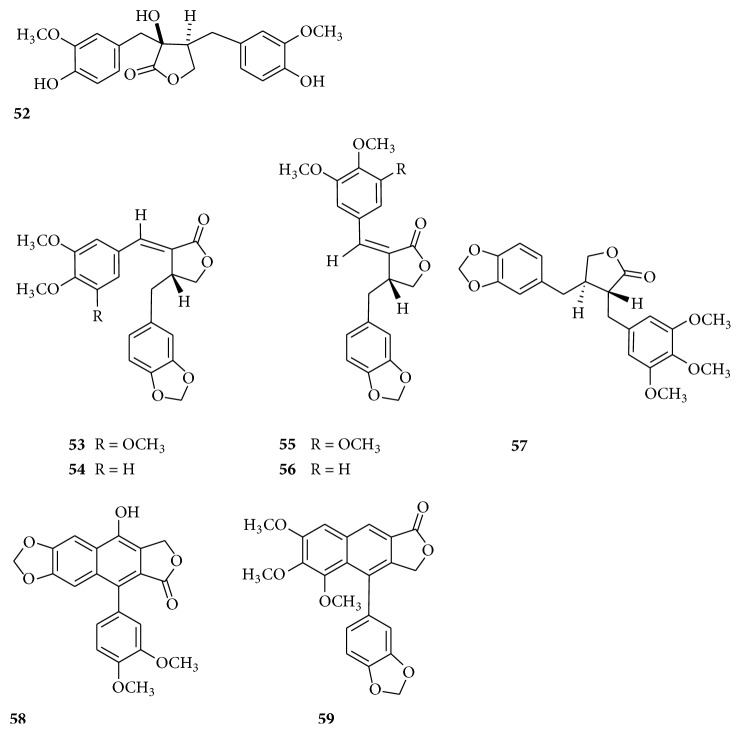
Chemical structures of lignans.

**Figure 6:**
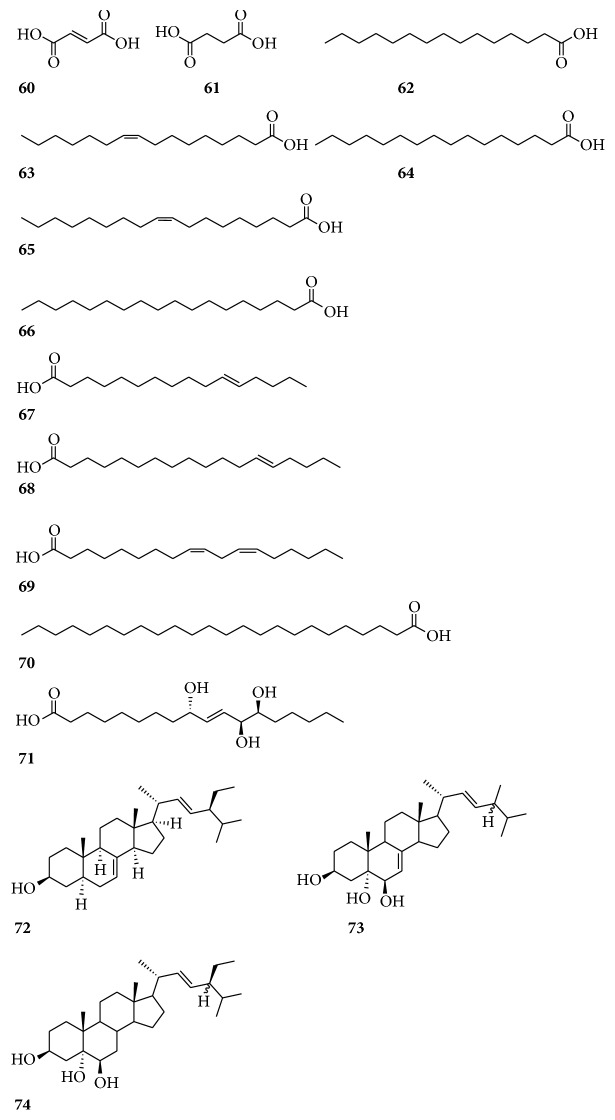
Chemical structures of other compounds.

In the past few decades, approximately 74 compounds have been isolated from* Radix Bupleuri*, including essential oils, triterpenoid saponins, polyacetylenes, flavonoids, lignans, fatty acids, and sterols. Triterpenoid saponins, flavonoids, and essential oil, which possess multiple pharmacological activities, are considered as the main active ingredients of* Radix Bupleuri*. Their structures are shown in (Table 2) (Figures 2–6).

### 4.1. Volatile Compounds (Essential Oils)

The essential oils of* Radix Bupleuri *are regarded as one of the most important bioactive compounds owing to their antifungal and anti-inflammatory activities [37, 38]. In one study, the essential oils in* Radix Bupleuri *were extracted by steam distillation and solvent extraction and then analyzed by GC/MS; 78 peaks were identified. Among these peaks, the major volatile compounds were 3-methylbutanal (7.24%), pentanal (5.74%), hexanal (20.11%), furan-2-carbaldehyde (25.23%), and heptanal (12.07%) [39]. However, in another study, the results showed that E-2-heptanal, furan, 2-pentyl, and E-2-nonenal were some of the main compounds of the oil [40].

### 4.2. Triterpenoid Saponins

Triterpenoid saponins are the main active components of* Radix Bupleuri*, which exhibit a broad spectrum of biological and pharmacological effects, including analgesic, immunomodulatory, hepatoprotective, immunomodulatory, anti-inflammatory, antitumor, and antiviral activities [3, 41–43]. Currently, approximately 35 saponins have been isolated from* Radix Bupleuri* (Figure 2) [44–54]. Among them, saikosaponins a, c, and d are the major bioactive constituents found in* Radix Bupleuri*; however a variety of minor saikosaponins have also been isolated [16]. The cytotoxic and antiproliferative effects of saikosaponins a and d have been attracting much interest in recent years [55]. Additionally, more information about the intimate relationship between the structural characterization of saikosaponins and their cytotoxic evaluations is very necessary.

### 4.3. Polyacetylenes

Four polyacetylene compounds from* Radix Bupleuri* have been identified, including (2Z,8Z,10E)-pentadecatriene-4,6-diyne-1-ol (**36**), (2Z,8E,10E)-pentadecatriene-4,6-diyne-1-ol (**37**), (2Z,8Z,10E)-heptadecatriene-4,6-diyne-1-ol (**38**), and bupleurynol (**39**) (Figure 3) [56, 57].

### 4.4. Flavonoids

So far, twelve flavonoids have been isolated and identified from* Radix Bupleuri*, including quercetin (**40**), isorhamnetin (**41**), isorhamnetin-3-O-glucoside (**42**), puerarin (**43**), rutin (**44**), narcissin (**45**), eugenin (**46**), saikochrome A (**47**), saikochromic acid (**48**), 7,4′-dihydroxy-isoflavone-7-O-*β*-D-glucoside (**49**), saikochromoside A (**50**), and saikoisoflavonoside A (**51**) (Figure 4) [58–63].

### 4.5. Ligans

Lignans existing in numerous plants were detected in the roots of* Bupleurum scorzonerifolium *Willd. These lignans included nortrachelogenin (**52**), nemerosin (**53**), kaerophyllin (**54**), isochaihulactone (**55**), isokaerophyllin (**56**), (−)-yatein (**57**), chinensinaphthol (**58**), and chaihunaphthone (**59**) (Figure 5) [62, 63].

### 4.6. Other Compounds

There are also other components in* Radix Bupleuri*, including 12 fatty acids: fumaric acid (**60**), butanedioic acid (**61**), pentadecanoic acid (**62**), palmitoleic acid (**63**), palmitic acid (**64**), oleic acid (**65**), stearic acid (**66**), 11-hexadecenoic acid (**67**), 13-octadecenoic acid (**68**), linoleic acid (**69**), tetracosanoic acid (**70**), and 9S,12S,13S-trihydroxy-10E-octadecenoic acid (**71**). Three sterols compounds, namely, *α*-spinasterol (**72**), 24*ξ*-methylcholesta-7, 22E-diene-3*β*,5*α*,6*β*-triol (**73**), and 24*ξ*-ethylcholest-22E-end-3*β*,5*α*,6*β*-triol (**74**) were also isolated and identified from this plant (Figure 6) [63–65].

## 5. Pharmacological Effects

*Radix Bupleuri *exerts a great variety of pharmacological activities due to its complexbioactive compounds. An overview of the pharmacological studies on* Radix Bupleuri*is presented in detail in the following sections.

### 5.1. Anti-inflammatory Effects

*Radix Bupleuri* has been widely used for the treatment of several types of chronic inflammatory diseases. The crude polysaccharides (80 mg/kg) isolated from the roots of* Bupleurum chinense* DC. significantly attenuated lung injury by inhibiting the level of myeloperoxidase (MPO), tumor necrosis factor-*α* (TNF-*α*), and serum nitric oxide (NO) [66]. Chun et al. reported that saikosaponins from* Radix Bupleuri* exhibited anti-inflammatory activity on inflammatory processes including inhibition of inflammatory exudation, capillary permeability, inflammatory mediators release, migration of white cells, connective tissue hyperplasia, and a variety of allergic inflammation [67]. Ma et al. was the first to show that saikosaponins exerted anti-inflammatory activity on paw edema mainly via regulating the nicotinate and nicotinamide metabolism and arachidonic acid metabolism [5]. Zhu et al. found that saikosaponin a (SSa) exhibited an inhibitory effect on proinflammatory cytokines in LPS-stimulated macrophages. The mechanism of these actions involved the regulation of MAPK and NF-*κ*B signals pathways [68]. In another study, SSa dose-dependently inhibited the production of ROS, TNF-*α*, IL-8, COX-2, and iNOS in LPS-stimulated human umbilical endothelial cells (HUVECs) by upregulating of the LXR*α*-ABCA1 signaling pathway [69]. Moreover, Lee et al. showed that saikosaponin c (SSc) was also shown to inhibited LPS-induced apoptosis in HUVECs via inhibition of caspase-3 activation and caspase-3-mediated-FAK degradation [70]. Zhao et al. showed that SSa also suppressed TNF-*α* and IL-6 concentrations in the intestines of septic rats through the inhibition of the nucleotide-binding oligomerization domain 2 (NOD2)/NF-*κ*B signaling pathway [71]. Saikosaponin d (SSd) has been reported to inhibit PGE_2_ production and intracellular free Ca^2+^ concentration ([Ca^2+^]i) in a concentration-dependent manner with an IC_50_ value of 3 *μ*m in C6 rat glioma cells [72].

In addition, several studies showed that a wide range of* Radix Bupleuri* preparations also exhibited anti-inflammatory effects in in vitro and in vivo model. Li et al. showed that Saireito and its active components (SSd) could suppress the proliferation of mesangial cells and expansion of the mesangial matrix in the rat glomerulonephritis model [73]. In experimental chronic pancreatitis rats model, “Chai-hu-shu-gan powder” exerted anti-inflammatory and antifibrotic effects by inhibiting the expression of nuclear factor-*κ*B (NF-*κ*B) and TNF-*α* mRNA in the pancreas [74]. Furthermore, it also reduced the abnormally high plasma level of cholecystokinin, improved the gastric movement, and avoided nausea and flatulence [75]. In another experiment, a Chinese herbal formula called “RCM-101” (containing* Flos magnoliae*,* Radix Bupleuri*,* Radix Glycyrrhizae*,* Radix Angelicae Sinensis*, etc.) inhibited the NO production and iNOS protein expression in LPS-stimulated rat aorta and Raw 264.7 macrophages [76].

### 5.2. Anticancer Effects

The extracts and compounds of* Radix Bupleuri* also possessed anticancer/antitumor effect. The acetone extract of* Bupleurum scorzonerifolium* could inhibit the proliferation of A549 human lung cancer cells in a dose-dependent manner via causing cell cycle arrest in the G2/M phase, increasing microtubule stabilization, suppressing telomerase activity, activating ERK 1/2 and caspase-3/9 in A549 cells [77–79]. Saponins isolated from* Radix Bupleuri* also exhibited significantly anti-proliferative activity in human non-small cell lung cancer A549 cells through Fas-dependent apoptotic pathway [80]. Su et al. found that the water extracts of* Radix Bupleuri *could enhance 5-fluorouracil-induced cytotoxicity in HepG2 hepatoma cells through cell arrest at the late G1/early S phase, while protecting normal blood lymphocytes [6]. SSd showed very potent activity against the HepG2 cell line with an IC_50_ value of 12.5 mg/ml. The mechanism of cytotoxicity was attributed to the induction of apoptosis through activation of caspase-3 and caspase-7, which subsequently resulted in poly-ADP-ribose polymerase (PARP) cleavage [81]. The study by Hou et al. demonstrated that SSd exerted its antitumor effect in human hepatocellular carcinoma cell line SMMC-7721 through the HIF-1*α*/COX-2 pathway [82]. Furthermore, SSd was also reported to induce autophagy in HeLa and MCF-7 cancer cells by direct inhibition of sarcoplasmic/endoplasmic reticulum Ca^2+^ ATPase (SERCA), leading to the increase of intracellular calcium ion levels and activating the Ca^2+^/calmodulin-dependent kinase kinase-*β*- (CaMKK*β*-) AMP-activated protein kinase- (AMPK-) mammalian target of rapamycin (mTOR) signaling cascade, endoplasmic reticulum (ER) stress, and unfolded protein responses (UPR) [43].

Several Chinese medicine preparations containing* Radix Bupleuri* also have been traditionally used in the treatment of tumors and cancer. The water extracts of “Long Dan Xie Gan Wan” exerted a significant growth inhibitory effect in HL60 and HT29 cancer cell lines, indicating that this formulation may possess some chemotherapeutic potential [83]. Treatment with “xiao-chai-hu decoction” exhibited a significantly lower incidence of hepatocellular carcinoma and reductions in cancer pain and tumor size. The underlying mechanism of the antitumor activities is based on stimulation of the reticuloendothelial system (RES) and is closely related of TNF production [84–86].

### 5.3. Antiviral Effects

Wen et al. reported that the acetone extract of* Radix Bupleuri* possessed a significant antivirus effect on acute respiratory tract infections with H1N1 virus infection and suppressed influenza A virus-induced RANTES secretion in H1N1-infected A549 cells at a concentration of 100 and 200 *μ*g/ml, suggesting that* Radix Bupleuri* might be beneficial for the treatment of chronic inflammatory conditions followed by viral infection [10]. SSc has been reported to show effective anti-HBV activity through inhibiting DNA expression of HBsAg, HBeAg, and HBV [81]. Treatment with “xiao-chai-hu decoction” (20 *μ*g/ml, 3 days; 20 *μ*g/ml, 6 days) could inhibit the production of HBV (*P* < 0.0001) and the expression of HBeAg. Moreover, crude saponins of* Bupleurum chinense* DC. could inhibit the replication of HBV (*P* < 0.0001) [87]. Similarly, in another study, Yin et al. showed that SSd isolated from the MeOH extract of* Bupleurum chinense* DC. exhibited significant bioactivity in inhibiting DNA replication of HBV [88]. The antiviral activity of saikosaponins (a, b_2_, c, and d) and their mode of actin were examined. The results showed that all saikosaponins exerted antiviral activity on human coronavirus-229E at concentrations of 0.25–25 *μ*m, and the strongest activity was observed for saikosaponin b_2_ with an IC_50_ of 1.7 *μ*m. This mechanism might involve interference in the early stage of viral replication, such as absorption and penetration of the virus [89].

### 5.4. Antipyretic Effects

The water extract of* Radix Bupleuri* was reported to exert its antipyretic effect on dry yeast-induced high fever rats. The mechanism is related to the adjustment of synthesis and exudation of cyclic adenosine monophosphate (cAMP) and arginine vasopressin (AVP) [90]. A novel in situ gel system for nasal delivery of the essential oil from* Radix Bupleuri* was prepared. The results suggested that* Radix Bupleuri* in situ gel can be more effective than the solution in the treatment of fever [91]. A similar study showed that the essential oil extracted from the herb exhibited dose-dependent antipyretic capacity on both fevered rabbits and rats [92].

### 5.5. Antibacterial Effects

The ethanol extract of* Bupleurum chinense* DC. exerted a remarkable bacteriostatic effect on Gram-negative microorganism* Helicobacter pylori*. The bioactive minimum inhibitory concentration (MIC) value was 60 Mm [93]. Saikosaponins isolated from* Radix Bupleuri *have been reported to exhibit antibacterial activity, particularly against* Pseudomonas aeruginosa* and* Listeria monocytogenes*. The protective effect was attributed to the immunomodulatory action on macrophages [94]. “Chaihu injection” has also been tested for possible antimicrobial activity in vitro. The results demonstrated that mild inhibition of* Staphylococcus aureus* was observed but no effects were observed against* Staphylococcus albus*,* Neisseria gonorrhoeae*,* Diplococcus pneumoniae*,* haemolytic Streptococcus, *or* Pseudomonas aeruginosa* [95].

### 5.6. Hepatoprotective Effects

The liver protective effects against CCl_4_ induced liver injury were investigated after treatment of mice with raw* and *vinegar-baked* Radix Bupleuri* (5 g/kg/day) for 14 days. The results showed that both raw and processed* Radix Bupleuri* showed liver protective effects against CCl_4_ induced liver injury, and the vinegar-baked* Radix Bupleuri* exerted better effects than that of raw* Radix Bupleuri* [96]. Pretreated with saikosaponins, especially SSa or SSd, showed remarkable inhibition of D-galactosamine-induced hepatic injury through decreasing the activity of glucose-6-phosphatase and NADPH-cytochrome C reductase and increasing 5′-nucleotidase activity [97]. Similarly, bupleurosides III, VI, IX, and XIII and saikosaponin b_3_ isolated from* Bupleurum scorzonerifolium *Willd. were also found to exhibit protective effect on the D-galactosamine-induced cytotoxicity in primary cultured rat hepatocytes [98]. Further studies also demonstrated that the protective effects of saikosaponins isolated from* Bupleurum chinense* DC. could prevent hepatocyte injury through regulating intracellular calcium levels [99]. In a rat model with CCl_4_ induced acute hepatic injury, the hepatic enzyme levels (GOT, GPT, and ALP) and the lipid peroxidation in the liver were significantly reduced by the administration of SSd [100]. Additionally, SSd significantly reduced collagen I deposition and alanine aminotransferase level on liver fibrosis rats and decreased the concentration of transforming growth factor *β*1 (TGF-*β*1). Moreover, SSd was able to alleviate hepatocyte injury from oxidative stress. The effect of SSd on liver fibrosis may be related to its ability to reduce lipid peroxidation [101].

### 5.7. Immunomodulatory Effects

Yamakage et al. determined the effects of* Radix Bupleuri* on spontaneous lymphatic vessel activity. The results indicated that* Radix Bupleuri* significantly increased the amplitude of spontaneous activity of lymphatic vessels in a concentration-dependent manner, and the mechanisms of this effect seem to be independent of endothelial function [102]. Eugenin (**46**) and saikochrome A (**47**) isolated from the MeOH extracts from* Bupleurum scorzonerifolium* possessed immunosuppressive effect on human peripheral blood T cells via inhibiting CD28-costimulated activation [62]. SSd (10 mg, intraperitoneally) significantly activated peritoneal macrophages in terms of enhancement of phagocytic activity, increased level of cellular lysosomal enzyme, and suppressed the response of plaque-forming cells to heterologous erythrocytes by stimulating T and B cells in a dose-dependent manner [103]. Moreover, SSd modulated lymphocyte activity through suppressing the T cell response and increasing the B cell response to different mitogens and the interleukin- (IL-) 2/IL-4 production through a receptor-bypassed pathway [41, 104, 105]. In another experiment, Wong et al. found that SSd was shown to inhibit OKT3/CD28-costimulated human T cell proliferation and PMA, PMA/ionomycin, and Con A-induced mouse T cell activation in vitro. The underlying mechanisms involved downregulation of NF-kB signaling by suppression of IKK and Akt activities [106].

### 5.8. Autophagic Effect

Autophagy is a complex process in cells, which occurs through the formation of double-membrane vesicles (autophagosomes), which are engulfed by cytoplasmic molecules. Then, the autophagosome fuses with the lysosomes, leading to degradation of long-lived proteins, aggregated proteins, and damaged organelles [107–109]. Moreover, autophagy might be triggered by hypoxia, nutritional deprivation, radiation, chemical drugs, and other stimulants [110]. Autophagy contributes to the pathogenesis of diverse diseases, such as neuronal degeneration, inflammatory bowel disease, aging, and cancer [111, 112]. In the previous study, Law et al. demonstrated that the protective pharmacological effects of* Radix Bupleuri* might be attributed to its autophagy induction. The autophagic effect of* Radix Bupleuri *played an important role in relieving liver disease-related symptoms through anti-inflammatory, organ-protective, and aggregate removal functions. Furthermore, the anticancer effects of* Radix Bupleuri* could be attributed to its autophagy induction.* Radix Bupleuri* has been found to be an effective treatment against depression by regulating metabolite, hormone, and neurotransmitter levels via autophagy-mediated lipid metabolism [113].

### 5.9. Other Pharmacological Effects

The effect of the ethanol extract from* Radix Bupleuri *on cytochrome 450 isoform activities using a six-drug cocktail approach was evaluated; the results demonstrated that* Radix Bupleuri* had strong induction activity on the CYP2E1, CYP2D6, and CYP3A4, which may lead to potential plant drug-drug interactions [114].* Radix Bupleuri* was shown to be the inhibitor of *β*-glucuronidase. The inhibition rate of* Radix B*upleuri extracts RB1 (high molecular weight polysaccharides), RB2 (ethanol soluble/water insoluble component), RB3 (extracted by n-butanol, soluble in water), and RB4 (low molecular weight water soluble parts) on the activity of *β*-glucuronidase was found to be 45.15%, 33.94%, 24.94%, and 34.54%, respectively [115]. In pentylenetetrazol (PTZ) induced epilepsy rats model, SSa isolated from* Radix Bupleuri *significantly reduced seizure severity and duration while it markedly elevated seizure latency and downregulated the cytokines expression of p-mTOR, p-70S6K, L-1*β*, and TNF-*α* through inhibiting mTOR signaling pathway [116]. He et al. demonstrated that SSa obviously reduced lipoprotein uptake to block foam cell formation and the expression of LOX-1 and CD36, boosted cholesterol efflux, and the expression of ABCA1 and PPAR*γ* through inhibiting PI3K/Akt/NF-*κ*B/NLRP3 signaling pathway [117]. In another experiment, SSc exerted a potent effect on inducing human umbilical vein endothelial cells (HUVECs) viability and growth. Furthermore, SSc also induced endothelial cells migration and capillary tube formation. The underlying mechanisms might be related to the gene expression or activation of matrix metalloproteinase-2 (MMP-2), vascular endothelial growth factor (VEGF), and the p42/p44 mitogen-activated protein kinase (MAPK, ERK) [118]. In addition, SSc was shown to exhibit inhibitory activities against Alzheimer's disease (AD) via suppressing the secretion of A*β* peptides and abnormal tau hyperphosphorylation-mediated microtubule depolymerization. Moreover, SSc suppressed A*β* peptide-induced brain endothelial apoptosis, indicating that Ssc might be a novel therapeutic tool for treating human AD and other neurodegenerative diseases [119]. It was shown by Liu et al. for the first time that four polyacetylenes (**36–39**) from* Radix Bupleuri *potently exhibited an antidepressant activity by inhibiting the reuptake of serotonin, norepinephrine, and dopamine. The mechanism might be mediated by increasing the level of monoamines, particularly 5-HT and NE [56]. Zhu et al. suggested that SSa and SSd exhibited the anthelmintic activity against* Dactylogyrus *spp. infecting goldfish. The effective concentration (EC_50_) values for SSa and SSd were 1.46 and 0.74 mg^−1^, respectively [120].

### 5.10. Summary of Pharmacological Effects

*Radix Bupleuri *possesses a wide spectrum of pharmacological effects, including anti-inflammatory effect, anticancer effect, antiviral effect, antipyretic effect, antibacterial effect, hepatoprotective effect, and immunomodulatory effect (Table 3). Based on these pharmacological effects, we can conclude that the extracts and the compounds from this plant can prevent or treat certain diseases, such as cancer, fever, malaria, hepatitis, and AD. However, there is not enough systemic data of these chemical compounds and their pharmacological effects. Thus, in the future, the pharmacological effects and the possible molecular mechanisms of the pharmacological activities of* Radix Bupleuri* must be urgently explored on our modern understanding of these diseases' pathophysiologies.

## 6. Toxicology

*Radix Bupleuri *has been used for thousands of years as an important traditional herb in China. However, the toxic effects of* Radix Bupleuri* in clinical applications have been gradually reported. Several studies have found that the liver is the main organ affected by toxicity, particularly in long-term use. Major symptoms of liver injury induced by* Radix Bupleuri* included transaminase lifts, hepatitis, and jaundice. However, liver functions can return to normal levels after a specific period [121].* Radix Bupleuri* has been reported to exhibit acute hepatitis and acute hepatic necrosis. The mean total daily dose was 18.0 ± 33.5 g, which was more than the Chinese Pharmacopoeia recommended range of 3 to 10 g [122]. Moreover,* Radix Bupleuri* had been implicated in multiple cases of acute hepatitis both as an ingredient alone and within a particular formulation “Xiao-Chai-Hu-Tang” (also known as Syo-Saiko-To in Japanese) [123]. Lee et al. demonstrated that two Chinese herbal products containing* Radix Bupleuri* might increase their risks of liver injury in HBV-infected patients. However, further mechanistic research on the hepatotoxicity of* Radix Bupleuri* in the presence of HBV infection is warranted [124]. In addition, the essential oil of* Radix Bupleuri* induced acute hepatotoxicity with asynchronous state, higher heart rate, and fast breathing [125]. The total saponins isolated from* Radix Bupleuri* could also cause evidently liver damage in dose-dependent manner manifested as hepatocyte organic lesion and liver function changes, as well as hepatocyte death [126].

## 7. Pharmacokinetics

A selective and sensitive LC-MS/MS method was developed and validated for simultaneous determination of SSa, b_2_, c, and d in rat plasma afteroral administration of the ethanol-water (50 : 50, v/v) extract of* Radix Bupleuri* for the first time. The results demonstrated that SSa, c, and d were absorbed rapidly with *T*_max_ less than 30 min [127]. In another pharmacokinetics experiment of rats, Liu et al. was the first to develop an UPLC-PDA-MS method to determine the pharmacokinetics of four polyacetylenes after i.g. administration of 95% ethanol extract of* Radix Bupleuri*. The results showed that compounds** 36** and** 38** were not detected in rat serum, whereas compounds** 37 **and** 39 **exerted a fast distribution phase followed by a relatively slow elimination phase (*t*_1/2*z*_, 4–7 h) [56].

## 8. Future Perspectives and Conclusions

In traditional Chinese medicine,* Radix Bupleuri* has long been used regulate the exterior and interior metabolisms, disperse evil heat from superficies, sooth the liver, and promote yang and qi. It has been widely used to treat various diseases in China, Japan, Korea, and other Asian countries for many centuries. A total of 74 compounds including essential oils, triterpenoid saponins, polyacetylenes, flavonoids, lignans, fatty acids, and sterols have been isolated and identified from* Radix Bupleuri* [39–65]. Pharmacological studies have revealed that* Radix Bupleuri* possesses a variety of biological effects, including anti-inflammatory, anticancer, antiviral, antipyretic, antibacterial, antiobesity, immunomodulatory, hepatoprotective, neuroprotective, and autophagic effects [66–120]. However, there are some aspects that need to be further investigated.

*Radix Bupleuri *is an ingredient of many patent medicines or prescriptions. Although modern experiments have confirmed that this drug alone exhibits multiple pharmacological activities, it is important to investigate the molecular mechanisms of* Radix Bupleuri* combined with other herbs based on traditional uses. Furthermore, the pharmacological effects of only a few of the ingredients, such as saikosaponins, flavonoids, and the essential oils, have been investigated. Some polyacetylenes, lignans, and sterols have not been sufficiently researched in terms of their pharmacological effects.* Radix Bupleuri* shows both hepatoprotection and hepatotoxicity, which appears to be contradictory. This phenomenon is similar to that of* Polygonum multiflorum *Thunb. [128]. Based on the literature, the main reasons are likely the administration dosage and delivery time. High doses and long-term drug delivery are more likely to result in liver toxicity, whereas low doses and short-term drug delivery might result in liver protection. Therefore, this issue needs further study.

In conclusion, this review summarized the traditional uses, botany, phytochemistry, pharmacology, and toxicology of* Radix Bupleuri*. Moreover, it has provided a new foundation for further research on its mechanism of action and the development of better therapeutic agents employing* Radix Bupleuri *in the future. It is anticipated that the comprehensive and detailed research on toxicity, pharmacodynamics, pharmacokinetics, and molecular mechanism are necessary to be explored to develop its bioactive compounds as effective drugs.

## Figures and Tables

**Table 1:** The traditional and clinical uses of *Radix Bupleuri *in China.

Preparation name	Compositions	Traditional uses
Xiao Chai Hu decoction	Radix Bupleuri, Radix Scutellariae, Panax ginseng, *Pinellia ternata*, Radix Glycyrrhizae, Rhizoma Zingiberis Recens, Fructus jujubae.	Curing thoracic and hypochondrium fullness, mouth-bitterness, throat-drying, and eyes-dazzling
Da Chai Hu decoction	Radix Bupleuri, Radix Scutellariae, *Pinellia ternata*, Rhizoma Zingiberis Recens, Fructus jujubae, Fructus Aurantii immaturus, Radix Paeoniae Alba, *Rheum palmatum*.	Treating diarrhea or constipation
Chaihu Guizhi decoction	Radix Bupleuri, Radix Scutellariae, Panax ginseng, *Pinellia ternata*, Radix Glycyrrhizae, Rhizoma Zingiberis Recens, Fructus jujubae, Radix Paeoniae Alba, Ramulus cinnamomi.	Curing fever with cold aversion, restless arthralgia of limbs, and epigastric induration
Chaihu Guizhi Ganjiang decoction	Radix Bupleuri, Ramulus cinnamomi, Rhizoma Zingiberis, Radix Scutellariae, Radix Glycyrrhizae, *Trichosanthes kirilowii*, Ostreae Concha.	Curing palpitation, fullness of the upper abdomen, and a bitter taste in the mouth
Chaihu plus Mangxiao decoction	Radix Bupleuri, Radix Scutellariae, Panax ginseng, *Pinellia ternata*, Radix Glycyrrhizae, Rhizoma Zingiberis Recens, Fructus jujubae, Natrii Sulfas.	Curing thoracic and hypochondrium fullness, retching counterflow, hot flashes, mouth-bitterness, throat-drying, eyes-dazzling, and constipation
Chaihu plus Longgu Muli decoction	Radix Bupleuri, Radix Scutellariae, Panax ginseng, *Pinellia ternata*, Rhizoma Zingiberis Recens, Fructus jujubae, *Rheum palmatum*, Ramulus cinnamomi, Ostreae Concha, Os Draconis, Poria cocos, Minium.	Curing thoracic and hypochondrium fullness, difficulty in micturition, and delirium
Buzhong Yiqi decoction	Radix Astragali, Radix Bupleuri, Panax ginseng, Radix Glycyrrhizae, Rhizoma Cimicifugae, Radix Angelicae sinensis, Atractylodis Macrocephalae Rhizoma, Citri Reticulatae Pericarpium.	Curing prolapse of uterus, prolapse of rectum, uterine bleeding, shortness of breath and tiredness, and pale tongue
Chaige Jieji decoction	Radix Bupleuri, Radix Puerariae lobatae, Radix Glycyrrhizae, Radix Scutellariae, Rhizoma et Radix Notopterygii, Radix Angelicae dahuricae, Radix Paeoniae Alba, Radix Platycodonis, Gypsum fibrosum, Rhizoma Zingiberis Recens, Fructus jujubae.	Curing mind-cold due to exogenous evils, headache, and vexation and sleeplessness
Zheng Chaihu Yin	Radix Bupleuri, Pericarpium Citri Reticulatae, Radix Saposhnikovia, Radix Paeoniae Rubra, Radix Glycyrrhizae, Rhizoma Zingiberis Recens.	Curing mind-cold due to exogenous evils, fever with chilliness, headache, and body pain
Xuefu Zhuyu decoction	Radix Angelicae sinensis, Radix Rehmanniae, Semen Persicae, Flos Carthami, Fructus Aurantii, Radix Bupleuri, Radix Glycyrrhizae, Radix Platycodonis, Rhizoma Chuanxiong, Radix Achyranthis Bidentatae, Radix Paeoniae Rubra	Treating blood pressure related symptoms caused by qi stagnation and blood stasis syndrome
Shengxian Decoction	Radix Astragali, Radix Bupleuri, Rhizoma Anemarrhenae, Radix Platycodonis, Rhizoma Cimicifugae.	Curing coronary heart disease, chronic congestive heart failure, vital myocarditis, and myocardial ischemia
Xiaoyao san	Radix Bupleuri, Radix Angelicae Sinensis, Radix Paeoniae Alba, Rhizoma Atractylodis Macrocephalae, Poria, Radix Glycyrrhizae, Herba Menthae, and Rhizoma Zingiberis Recens.	Soothing the liver and improving the circulation of qi to relieve depression
Chaihu Shugan San	Radix Bupleuri, Fructus Aurantii, Rhizoma Chuanxiong, Rhizoma Cyperi, Pericarpium Citri Reticulatae, Radix Paeoniae Rubra, Radix Glycyrrhizae.	Curing neurologic impairment, and depression
Sini San	Radix Bupleuri, Fructus Aurantii, Radix Paeoniae Rubra, Radix Glycyrrhizae.	Curing insomnia, liver injury, hepatitis, chronic stress model, and palmoplantar hidrosis
Tongqi San	Radix Bupleuri, Rhizoma Cyperi, Rhizoma Chuanxiong.	Curing deafness, tinnitus, and stagnation of liver qi
Danzhi Xiaoyao San	Radix Bupleuri, Radix Angelicae Sinensis, Radix Paeoniae Alba, Rhizoma Atractylodis Macrocephalae, Poria, Cortex Moutan, Fructus Gardeniae.	Curing headache, menoxenia, distending pain in the lower abdomen, and depression
Yigan Yiqi Jieyu Keli	Radix Bupleuri, Fructus Aurantii, Radix Paeoniae Alba, Radix et Rhizoma Salviae miltiorrhizae, Radix Astragali, Radix Codonopsis, Ramulus cinnamomi, Poria, Acanthopanax senticosus (Rupr. et Maxim) Harms, Fructus trichosanthis, Rhizoma Pinelliae Preparatum, and others.	Curing chronic hepatitis, hypochondriac pain, bloating, and lassitude
Xiao' er Tuire Keli	Radix Bupleuri, Folium isatidis, Radix Isatidis, Flos Lonicerae Japonicae, Cortex Moutan, Cape Jasmine Fruit, Radix Scutellariae, Herba Lophatheri, Pheretima, Rhizoma paridis, Radix et Rhizoma Cynanchi Atrati.	Curing cold due to exogenous wind-heat in children
Dalitong Keli	Radix Bupleuri, Fructus Aurantii, Radix Aucklandiae, Pericarpium Citri Reticulatae, Pinelliae Rhizoma Praeparatum Cum Alumine, Herba Taraxaci, Crataegi fructus, Semen Arecae, Paederia scandens, Radix Codonopsis, Rhizoma Corydalis, Massa Medicata Fermentata.	Curing epigastric fullness, belching, dry mouth, and mouth pain
Bubai Keli	Fructus Psoraleae, Dolichos lablab L., Folium Epimedii, Radix Bupleuri, Radix et Rhizoma Salviae miltiorrhizae, Vigna umbellata Ohwi et Ohashi, Radix Sophorae Flavescentis, Glycine max (L.) Merr.	Nourishing the spleen and warming the kidneys
Ganmao Qingre Keli	Schizonepetae Spica, Radix Bupleuri, Herba menthae haplocalycis, Radix Saposhnikovia, Perillae Folium, Radix Puerariae lobatae, Radix Platycodonis, Semen Armeniacae amarum, Radix Angelicae dahuricae, Corydalis bungeana Herba.	Curing cold, cough, fever, headache, rhinorrhea, and pharyngoxerosis
Qizhi Weitong tablet	Radix Bupleuri, Rhizoma Corydalis, Rhizoma Cyperi, Fructus Aurantii, Radix Paeoniae Alba, Radix Glycyrrhizae.	Curing stagnation of liver qi, abdominal distention, and epigastric pain
Lidan tablet	*Rheum palmatum*, Flos Lonicerae. Japonicae, Herba Lysimachiae, Radix Aucklandiae, Rhizoma Anemarrhenae, Folium isatidis, Radix Bupleuri, Radix Paeoniae Alba, Radix Scutellariae, Natrii Sulfas, Herba Artemisiae Scopariae.	Curing hypochondriac pain, constipation, oliguria with reddish urine, headache, and vomiting
Ruhe Sanjie tablet	Radix Bupleuri, Radix Angelicae sinensis, Radix Astragali, Radix Curcumae, *Tulipa edulis*, Radix Rhapontici, Ecklonia kurome Okam, Sargassum, Folium Epimedii, Herba Pyrolae.	Curing lump in breast, and mammary gland hyperplasia
Jianwei Yuyang tablet	Radix Bupleuri, Radix Paeoniae Alba, Radix Codonopsis, Rhizoma Corydalis, *Bletilla striata* (Thunb.) Reiehb. f., Indigo Naturalis, Concha Margaritifera Usta.	Curing dysphoria, distention and loose stools, and peptic ulcer
Longdan Xiegan pill	Radix et Rhizoma Gentianae, Radix Bupleuri, Radix Scutellariae, Fructus Gardeniae, Rhizoma alismatis, Caulis Akebiae, Semen Plantaginis, Radix Angelicae sinensis, Radix Rehmanniae, Radix Glycyrrhizae.	Curing hepatochlic hygropyrexia, dizziness, deafness, tinnitus, and mouth-bitterness
Qingwen Jiedu pill	Radix Bupleuri, Folium isatidis, Fructus Forsythiae, Radix Scrophulariae, Radix Trichosanthis, Radix Platycodonis, Fructus Arctii, Radix Saposhnikovia, Radix Puerariae lobatae, Radix Scutellariae, Radix Angelicae dahuricae, Rhizoma Chuanxiong, and others.	Curing headache, throat-drying, and mumps
Shugan Hewei pill	Rhizoma Cyperi, Radix Paeoniae Alba, Fructus citri Sarcodactylis, Radix Aucklandiae, Radix Curcumae, Rhizoma Atractylodis Macrocephalae, Pericarpium Citri Reticulatae, Radix Bupleuri, Herba Pogostemonis, Radix Glycyrrhizae, Semen Raphani, and others.	Curing disharmony between liver and stomach, epigastric pain, inappetence, and vomiting
Huayu Quban capsule	Radix Bupleuri, Radix Scutellariae, Radix Angelicae sinensis, Herba menthae haplocalycis, Flos Carthami, Radix Paeoniae Rubra	Curing chloasma and acne
Ruzengning capsule	Radix Bupleuri, Folium Artemisiae Argyi, Folium Epimedii, Fructus Toosendan, Radix Asparagi, Rhizoma bolbostemmatis.	Curing lump in breast and mammary gland hyperplasia
Mangan Jieyu capsule	Radix Bupleuri, Radix Angelicae sinensis, Radix Paeoniae Alba, Atractylodis Macrocephalae Rhizoma, Poria, Radix Glycyrrhizae, Herba menthae haplocalycis, Radix et Rhizoma Salviae miltiorrhizae, Fructus Toosendan, Fructus citri, Rhizoma Corydalis, and others.	Curing chest congestion, inappetence, abdominal distention, and chronic hepatitis
Biyuanshu capsule	Radix Bupleuri, Radix Scutellariae, Herba menthae haplocalycis, Radix Angelicae dahuricae, Flos Magnoliae, Fructus Xanthii, Fructus Gardeniae, Radix et Rhizoma Asari, Rhizoma Chuanxiong, Radix Astragali, Radix Platycodonis, Poria, Caulis Akebiae.	Curing rhinitis and nasosinusitis

**Table 2:** Chemical compounds isolated from *Radix Bupleuri*.

Classification	Number	Chemical component	Reference
Saponins	**1**	Saikosaponin a	[44]
	**2**	Saikosaponin c	[44]
	**3**	Saikosaponin d	[44]
	**4**	Saikosaponin e	[45]
	**5**	Prosaikogenin G	[46]
	**6**	Prosaikogenin F	[46]
	**7**	2′′-O-Acetylsaikosaponin a	[47]
	**8**	3′′-O-Acetylsaikosaponin a	[48]
	**9**	6′′-O-Acetylsaikosaponin a	[49]
	**10**	23-O-Acetylsaikosaponin a	[50]
	**11**	6′′-O-Acetylsaikosaponin d	[51]
	**12**	23-Hydroxy-13*β*, 28*β*-epoxy-olean-11-ene-16-one 3-O-*β*-D-glucopyranosyl-(1→3)-*β*-D-fucopyranoside	[52]
	**13**	3*β*,16*β*-Dihydroxy-23-O-acetyl-13*β*, 28*β*-epoxy-olean-11-ene 3-O-*β*-D-fucopyranoside	[47]
	**14**	Bupleuroside I	[45]
	**15**	Saikosaponin b_1_	[45, 49]
	**16**	Saikosaponin b_2_	[45, 49]
	**17**	6′′-O-Acetyl-saikosaponin b_2_	[51]
	**18**	Saikosaponin h	[47, 53]
	**19**	Prosaikogenin D	[54]
	**20**	Prosaikogenin A	[47]
	**21**	3*β*,23,28-Trihydroxy-11, 13(18)-diene-16-one 3-O-*β*-D-glucopyranosyl-(1→ 3)-*β*-D-fucopyranoside	[47]
	**22**	Bupleuroside V	[45]
	**23**	Bupleuroside X	[45]
	**24**	Bupleuroside XII	[45]
	**25**	Saikosaponin v-1	[51]
	**26**	Saikosaponin b_3_	[44, 53]
	**27**	Saikosaponin b_4_	[44, 53]
	**28**	Saikosaponin f	[45]
	**29**	3*β*,16*β*,23,28-Tetrahydroxy-11*α*-methoxy-olean-12-ene 3-O-*β*-D-fucopyranoside	[47]
	**30**	3*β*,16*β*,28-Trihydroxyl-11*α*-methoxy-olean-12-ene-O-*β*-D-fucopyranoside	[47]
	**31**	Bupleuroside VII	[45]
	**32**	Saikosaponin g	[53]
	**33**	Saikosaponin i	[53]
	**34**	Bupleuroside VIII	[45]
	**35**	Bupleuroside XI	[45]

Polyacetylenes	**36**	(2Z,8Z,10E)-pentadecatriene-4,6-diyne-1-ol	[56]
	**37**	(2Z,8E,10E)-pentadecatriene-4,6-diyne-1-ol	[56, 57]
	**38**	(2Z,8Z,10E)-heptadecatriene-4,6-diyne-1-ol	[56]
	**39**	Bupleurynol	[56, 57]

Flavonoids	**40**	Quercetin	[60]
	**41**	Isorhamnetin	[60]
	**42**	Isorhamnetin-3-O-glucoside	[60]
	**43**	Puerarin	[60]
	**44**	Rutin	[60]
	**45**	Narcissin	[59]
	**46**	Eugenin	[62]
	**47**	Saikochrome A	[62, 63]
	**48**	Saikochromic acid	[60]
	**49**	7,4′-Dihydroxy-isoflavone-7-O-*β*-D-glucoside	[60]
	**50**	Saikochromoside A	[59]
	**51**	Saikoisoflavonoside A	[61]

Lignans	**52**	Nortrachelogenin	[63]
	**53**	Nemerosin	[62]
	**54**	Kaerophyllin	[62]
	**55**	Isochaihulactone	[62]
	**56**	Isokaerophyllin	[62]
	**57**	(−)-yatein	[62]
	**58**	Chinensinaphthol	[62]
	**59**	Chaihunaphthone	[62]

Fatty acids	**60**	Fumaric acid	[64]
	**61**	Butanedioic acid	[64]
	**62**	Pentadecanoic acid	[65]
	**63**	Palmitoleic acid	[65]
	**64**	Palmitic acid	[65]
	**65**	Oleic acid	[65]
	**66**	Stearic acid	[65]
	**67**	11-Hexadecenoic acid	[65]
	**68**	13-Octadecenoic acid	[65]
	**69**	Linoleic acid	[65]
	**70**	Tetracosanoic acid	[64]
	**71**	9S,12S,13S-Trihydroxy-10E-octadecenoic acid	[63]

Sterols	**72**	*α*-Spinasterol	[64]
	**73**	24*ξ*-Methylcholesta-7, 22E-diene-3*β*,5*α*,6*β*-triol	[63]
	**74**	24*ξ*-Ethylcholest-22E-end-3*β*,5*α*,6*β*-Triol	[63]

**Table 3:** Pharmacological effects of *Radix Bupleuri*.

Pharmacological effects	Cell lines/model	Detail/mechanism(s) of action	Preparations/extracts/compounds	Application	Reference
Anti-inflammatory effect	LPS-induced mouse acute lung injury	Increases levels of MPO and TNF-*α*; Inhibits the No level	Crude polysaccharides	In vivo	[66]
	Formalin-induced mice paw edema	Regulates the nicotinate and nicotinamide metabolism and arachidonic acid metabolism	Saikosaponins	In vivo	[5]
	LPS-stimulated RAW 264.7 cells	Regulates MAPK and NF-*κ*B signals pathways	Saikosaponin a	In vitro	[68]
	LPS-stimulated human umbilical endothelial cells	Activates the LXR*α*-ABCA1 signaling pathway	Saikosaponin a	In vitro	[69]
	Septic rats model	Inhibits the nucleotide-binding oligomerization domain 2 (NOD2)/NF-*κ*B signaling pathway	Saikosaponin a	In vivo	[70]
	LPS-induced apoptosis in human umbilical vein endothelial cells	Inhibits the caspase-3 activation and caspase-3-mediated-FAK degradation	Saikosaponin c	In vitro	[71]
	C6 rat glioma cells	Inhibits PGE_2_ production and elevates intracellular free Ca^2+^ concentration	Saikosaponin d	In vitro	[72]
	Monoclonal antibody induced nephritis	Suppresses proteinuria, proliferation of mesangial cells, and expansion of the mesangial matrix	Saikosaponin d	In vivo	[73]
	Chronic pancreatitis rats model	Inhibits the expression of NF-*κ*B and TNF-*α* mRNA	Chai-hu-shu-gan powder	In vivo	[74]
	LPS-stimulated rat aorta and Raw 264.7 macrophages	Inhibits the NO production and iNOS protein expression	RCM-101	In vivo/in vitro	[76]

Anticancer effect	A549 human lung cancer cells	Suppresses telomerase activity and activates ERK 1/2 and caspase-3/9	Acetone extracts	In vitro	[77–79]
	Human non-small-cell lung cancer A549 cells	Regulates Fas-dependent apoptotic pathway	Saponins	In vitro	[80]
	HepG2 hepatoma cells	Induces cell arrest at the late G1/early S phase	Water extracts	In vitro	[6]
	HepG2 cell line	Activates caspase-3 and caspase-7	Saikosaponin d	In vitro	[81]
	SMMC-7721 cell line	Regulates the HIF-1*α*/COX-2 pathway	Saikosaponin d	In vitro	[82]
	HeLa and MCF-7 cancer cells	Inhibits the SERCA; Activates the CaMKK*β*-AMP-AMPK-mTOR signaling cascade, ER stress and UPR	Saikosaponin d	In vitro	[43]

Antiviral effect	H1N1-infected A549 cells	Anti-H1N1 virus	Acetone extracts	In vitro	[10]
	HBV-transfected human hepatoma cells	Inhibits DNA expression of HBsAg, HBeAg, and HBV	Saikosaponin c	In vitro	[81]
	HBV-transfected human hepatoma cells	Inhibits the production of HBV and the expression of HBeAg	Crude saponins	In vitro	[87]
	HBV-transfected human hepatoma cells	Inhibits DNA replication of HBV	MeOH extracts	In vitro	[88]
	Human coronavirus-229E	Interferes with the early stage of viral replication	Saikosaponins	In vitro	[89]

Antipyretic effect	Dry yeast-induced high fever rats	Adjusts the synthesis and exudation of cAMP and AVP	Water extracts	In vivo	[90]
	Turpentine-induced fever rabbits	Decreases body temperature	The essential oil	In vivo	[91]
	Turpentine-induced fever rabbits and rats	Decreases body temperature	The essential oil	In vivo	[92]

Antibacterial effect	*Helicobacter pylori* strain	Anti-*Helicobacter pylori*	Ethanol extracts	In vitro	[93]
	*Staphylococcus aureus* strain	Anti-*Staphylococcus aureus*	Chaihu injection	In vitro	[95]
	*Pseudomonas aeruginosa*, *Listeria monocytogenes* strains	Anti-*Pseudomonas aeruginosa*, Anti-*Listeria monocytogenes*	Saikosaponins	In vitro	[94]

Hepatoprotective effect	CCl_4_ induced hepatic damage in rats	Hepatoprotective effects	Methanol extracts	In vivo	[96]
	D-galactosamine-induced hepatic injury in rats	Decreases the activity of glucose-6-phosphatase and NADPH-cytochrome C reductase; increases 5′-nucleotidase activity	Saikosaponins	In vivo	[97]
	D-galactosamine-induced cytotoxicity in rat hepatocytes	Hepatoprotective effects	Bupleurosides III, VI, IX, and XIII; saikosaponin b_3_	In vitro	[98]
	BEL-7402 cells	Regulates intracellular calcium levels	Saikosaponins	In vitro	[99]
	CCl_4_ induced acute hepatic injury in rats	Hepatoprotective effects	Saikosaponin d	In vivo	[100]
	CR-1548 cell; liver fibrosis in rats	Reduces lipid peroxidation	Saikosaponin d	In vitro/in vivo	[101]

Immunomodulatory effect	Rats fed with* Radix Bupleuri*	Increases the amplitude of spontaneous activity of lymphatic vessels	Water extracts	In vivo	[102]
	Human peripheral blood T cells	Inhibits CD28-costimulated activation	MeOH extracts	In vitro	[62]
	BALB/c, C3H/He and CDF_1_ mice	Stimulates T and B cells	Saikosaponin d	In vivo	[103]
	Thymocytes and spleen cells; C57BL/6 mice	Regulates receptor-bypassed pathway	Saikosaponin d	In vitro/in vivo	[41, 104, 105]

Others	Male SD rats	Exhibits strong induction activity on the CYP2E1, CYP2D6, and CYP3A4	Ethanol extracts	In vivo	[107]
	PTZ-induced epilepsy rats model	Inhibits mTOR signaling pathway	Saikosaponin a	In vivo	[109]
	THP-1 Cells	Inhibits PI3K/Akt/NF-*κ*B/NLRP3 signaling pathway	Saikosaponin a	In vitro	[110]
	Human umbilical vein endothelial cells	Activates MMP-2, VEGF, and the p42/p44 mitogen-activated protein kinase	Saikosaponin c	In vitro	[111]
	SH-SY5Y and SK-N-SH cell lines	Suppresses the secretion of A*β* peptides	Saikosaponin c	In vitro	[112]
	Male Wistar rats	Increases the level of 5-HT and NE	Polyacetylenes	In vivo	[56]
	*Dactylogyrus* spp. infecting goldfish	Anthelmintic activity	Saikosaponins a and d	In vivo	[113]

## References

[1] (2015). *Editorial Committee of Chinese Pharmacopoeia, Chinese Pharmacopoeia*.

[2] Wang Y. S., Chang H. M., But P. P. H. (1987). *Pharmacology and applications of Chinese*.

[3] Ashour M. L., Wink M. (2011). Genus Bupleurum: a review of its phytochemistry, pharmacology and modes of action. *The Journal of Pharmacy and Pharmacology*.

[4] Bermejo B. P., Martinez M. J. A., Sen A. M. S. (1998). In vivo and in vitro antiinflammatory activity of saikosaponins. *Life Sciences*.

[5] Ma Y., Bao Y., Wang S. (2016). Anti-inflammation effects and potential mechanism of saikosaponins by regulating nicotinate and nicotinamide metabolism and arachidonic acid metabolism. *Inflammation*.

[6] Su J. K., Lee Y. J., Kim B. M. (2008). Effect of Bupleuri Radix Extracts on the Toxicity of 5-Fluorouracil in HepG2 Hepatoma Cells and Normal Human Lymphocytes. *Basic & Clinical Pharmacology & Toxicology*.

[7] Motoo Y., Sawabu N. (1994). Antitumor effects of saikosaponins, baicalin and baicalein on human hepatoma cell lines. *Cancer Letters*.

[8] Idris-Usman M. S. (2010). Antinociceptive and antipyretic properties of the pharmaceutical herbal preparation, Radix bupleuri in rats. *Journal of Medicinal Plants Research*.

[9] Zhou Q., Wang S.-S., Yang G., Zhao W., Li H.-L. (2016). Development and evaluation of a herbal formulation with anti-pathogenic activities and probiotics stimulatory effects. *Journal of Integrative Agriculture*.

[10] Wen S., Xu H. F., Hao H. (2011). In vitro anti-influenza A H1N1 effect of extract of Bupleuri Radix. *Immunopharmacology and Immunotoxicology*.

[11] Yoshikawa M., Murakami T., Hirano K., Inadzuki M., Ninomiya K., Matsuda H. (1997). Scorzonerosides A, B and C, novel triterpene oligoglycosides with hepatoprotective effect from Chinese Bupleuri radix, the roots of Bupleurum scorzonerifolium Willd.. *Tetrahedron Letters*.

[12] Wang Z., Li H., Xu H. (2009). Beneficial effect of Bupleurum polysaccharides on autoimmune disease induced by Campylobacter jejuni in BALB/c mice. *Journal of Ethnopharmacology*.

[13] Abe H., Sakaguchi M., Konishi H., Tani T., Arichi S. (1978). The effects of saikosaponins on biological membranes. *Planta Medica*.

[14] Pan S. L. (2006). Bupleurum species: scientific evaluation and clinical applications.

[15] Tian R.-T., Xie P.-S., Liu H.-P. (2009). Evaluation of traditional Chinese herbal medicine: Chaihu (Bupleuri Radix) by both high-performance liquid chromatographic and high-performance thin-layer chromatographic fingerprint and chemometric analysis. *Journal of Chromatography A*.

[16] Huang H. Q., Zhang X., Xu Z. X., Su J., Yan S. K. (2009). Fast determination of saikosaponins in Bupleurum by rapid resolution liquid chromatography with evaporative light scattering detection. *Journal of Pharmaceutical & Biomedical Analysis*.

[17] Sui C., Wei J.-H., Chen S.-L., Chen H.-Q., Dong L. M., Yang C.-M. (2010). Construction of a full-length enriched cDNA library and analysis of 3111 ESTs from roots of Bupleurum Chinense DC. *Botanical Studies*.

[18] Xie H., Huo K.-K., Chao Z., Pan S.-L. (2009). Identification of crude drugs from Chinese medicinal plants of the genus Bupleurum using ribosomal DNA ITS sequences. *Planta Medica*.

[19] Hsu L.-M., Huang Y.-S., Tsay S.-H., Chang F.-Y., Lee S.-D. (2006). Acute hepatitis induced by Chinese hepatoprotective herb, xiao-chai-hu-tang. *Journal of the Chinese Medical Association*.

[20] Van Wyk B. E., Wink M. (2004). *Medicinal Plants of the World: An Illustrated Scientific Guide to Important Medicinal Plants and Their Uses*.

[21] Xiong J. Y., Zang N., Zhang C. Y. (2007). Studies on the anti-depression effect of Xiaoyao Powder in mic. *Pharmacology and Clinics of Chinese Materia Medica*.

[22] Zhou Y., Ren Y., Ma Z. (2012). Identification and quantification of the major volatile constituents in antidepressant active fraction of xiaoyaosan by gas chromatography-mass spectrometry. *Journal of Ethnopharmacology*.

[23] Gao X., Zheng X., Li Z. (2011). Metabonomic study on chronic unpredictable mild stress and intervention effects of Xiaoyaosan in rats using gas chromatography coupled with mass spectrometry. *Journal of Ethnopharmacology*.

[24] Editorial Board of Flora of China (1998). *Flora of China*.

[25] Wang Y. Z., Zhang Y. Y. (1994). Determination of species of medical Bupleunum. *Chinese Pharmaceutical Journal*.

[26] Pan S. L., Shun Q. S., Bo Q. M., Bao X. S. (2002). *The coloured atlas of the medicinal plants from genus Bupleurum in China*.

[27] Yen M. H., Lin C. C., Chuang C. H., Liu S. Y. (1991). Evaluation of root quality of Bupleurum species by TLC scanner and the liver protective effects of “xiao-chai-hu-tang. *Journal of Ethnopharmacology*.

[28] Li X.-Q., Gao Q.-T., Chen X.-H., Bi K.-S. (2005). High performance liquid chromatographic assay of saikosaponins from Radix Bupleuri in China. *Biological and Pharmaceutical Bulletin*.

[29] Zhou Q., Hao J. P., Gao K. Q. (2012). Analysis on Saikosaponin of Sixteen Bupleurum chinense from Shanxi. *Natural Product Research & Development*.

[30] Tang Y.-H., Zhang Y.-Y., Zhu H.-Y., Huang C.-G. (2007). A high-performance liquid chromatographic method for saikosaponin a quantification in rat plasma. *Biomedical Chromatography*.

[31] Zhang H., Zhao Y., Liang H., Huang L., Zhang Q., Zhi X. (2007). Simultaneous HPLC-ELSD determination of saikosaponins a, c, d, f in Radix Bupleuri. *Chinese Journal of Pharmaceutical Analysis*.

[32] Lucena R., Cárdenas S., Valcárcel M. (2007). Evaporative light scattering detection: Trends in its analytical uses. *Analytical and Bioanalytical Chemistry*.

[33] Bao Y., Li C., Shen H., Nan F. (2004). Determination of saikosaponin derivatives in Radix bupleuri and in pharmaceuticals of the Chinese multiherb remedy Xiaochaihu-tang using liquid chromatographic tandem mass spectrometry. *Analytical Chemistry*.

[34] Liau B.-C., Hsiao S.-S., Lee M.-R., Jong T.-T., Chiang S.-T. (2007). Quality control of Chinese medicinal preparations. LC/ESI(+)/MS/MS analyses of saikosaponins-a and -c as markers of Bupleuri radix samples. *Journal of Pharmaceutical and Biomedical Analysis*.

[35] Chen Y., Wang J., Yuan L., Zhou L., Jia X., Tan X. (2011). Interaction of the main components from the traditional chinese drug pair chaihu-shaoyao based on rat intestinal absorption. *Molecules*.

[36] Lin X., Xue L., Zhang H., Zhu C. (2005). Determination of saikosaponins a, c, and d in Bupleurum Chinese DC from different areas by capillary zone electrophoresis. *Analytical and Bioanalytical Chemistry*.

[37] Fernandez-Ocana A. M., Gomez-Rodriguez M. V., Velasco-Negueruela A. (2004). In vivo antifungal activity of the essential oil of Bupleurum gibraltarium against Plasmopara halstedii in sunflower. *Journal of Agricultural & Food Chemistry*.

[38] Ocete M. A., Risco S., Zarzuelo A., Jimenez J. (1989). Pharmacological activity of the essential oil of Bupleurum gibraltaricum: Anti-inflammatory activity and effects on isolated rat uteri. *Journal of Ethnopharmacology*.

[39] Liu S. H., Lu S. S., Su Y. L., Guo Y. (2011). Analysis of volatile compounds in Radix bupleuri injection by GC-MS-MS. *Chromatographia*.

[40] Li X., Jia Y., Song A., Chen X., Bi K. (2005). Analysis of the essential oil from Radix Bupleuri using capillary gas chromatography. *Yakugaku Zasshi*.

[41] Ushio Y., Abe H. (1991). The effects of saikosaponin on macrophage functions and lymphocyte proliferation. *Planta Medica*.

[42] Ushio Y., Abe H. (1992). Inactivation of measles virus and herpes simplex virus by Saikosaponin d. *Planta Medica*.

[43] Wong V. K., Li T., Law B. Y. (2012). Saikosaponin-d, a novel SERCA inhibitor, induces autophagic cell death in apoptosis-defective cells. *Cell Death & Disease*.

[44] Otsuka H. (1978). Separation and determination of saponins of bupleuri radix by droplet counter current chromatography (DCC). *Planta Medica*.

[45] Matsuda H., Murakami T., Ninomiya K., Inadzuki M., Yoshikawa M. (1997). New hepatoprotective saponins, bupleurosides III, VI, IX, and XIII, from Chinese Bupleuri Radix: Structure-requirements for the cytoprotective activity in primary cultured rat hepatocytes. *Bioorganic and Medicinal Chemistry Letters*.

[46] Ding J.-K., Fujino H., Kasai R. (1986). Chemical evaluation of Bupleurum species collected in Yunnan, China. *Chemical and Pharmaceutical Bulletin*.

[47] Li D. Q., Wu J., Liu L. Y. (2015). Cytotoxic triterpenoid glycosides (saikosaponins) from the roots of Bupleurum chinense. *Bioorganic & Medicinal Chemistry Letters*.

[48] Seto H., Otake N., Luo S. Q., Qian F. G., Xu G. Y. (1986). Studies on chemical constituents of Bupleurum genus. Part II. Isolation of triterpenoid glycosides (saikosaponins) from Bupleurum kunmingense and their chemical structures. *Agricultural & Biological Chemistry*.

[49] Yang Y.-Y., Tang Y.-Z., Fan C.-L., Luo H.-T., Guo P.-R., Chen J.-X. (2010). Identification and determination of the saikosaponins in Radix bupleuri by accelerated solvent extraction combined with rapid-resolution LC-MS. *Journal of Separation Science*.

[50] Ishii H., Nakamura M., Seo S., Tori K., Tozyo T., Yoshimura Y. (1980). Isolation, characterization, and nuclear magnetic resonance spectra of new saponins from the roots of Bupleurum falcatum L.. *Chemical and Pharmaceutical Bulletin*.

[51] Liu Q.-X., Liang H., Zhao Y.-Y., Wang B., Yang W.-X., Yu Y. (2001). Saikosaponin v-1 from roots of Bupleurum chinense DC.. *Journal of Asian Natural Products Research*.

[52] Ogihara Y., Nose M. (1986). Novel cleavage of the glycosidic bond of saponins in alcoholic alkali metal solution containing a trace of water. *Journal of the Chemical Society, Chemical Communications*.

[53] Lee J., Yang D.-H., Suh J. H. (2011). Species discrimination of Radix Bupleuri through the simultaneous determination of ten saikosaponins by high performance liquid chromatography with evaporative light scattering detection and electrospray ionization mass spectrometry. *Journal of Chromatography B: Analytical Technologies in the Biomedical and Life Sciences*.

[54] Shimizu K., Amagaya S., Ogihara Y. (1985). New derivatives of saikosaponins. *Chemical and Pharmaceutical Bulletin*.

[55] Tundis R., Bonesi M., Deguin B. (2009). Cytotoxic activity and inhibitory effect on nitric oxide production of triterpene saponins from the roots of Physospermum verticillatum (Waldst & Kit) (Apiaceae). *Bioorganic and Medicinal Chemistry*.

[56] Liu J., Fang Y., Yang L., Qin X., Du G., Gao X. (2015). A qualitative, and quantitative determination and pharmacokinetic study of four polyacetylenes from Radix Bupleuri by UPLC-PDA-MS. *Journal of Pharmaceutical and Biomedical Analysis*.

[57] Huang H. Q., Su J., Zhang X., Shan L., Zhang W. D. (2011). Qualitative and quantitative determination of polyacetylenes in different *Bupleurum* species by high performance liquid chromatography with diode array detector and mass spectrometry. *Journal of Chromatography A*.

[58] Li Q. C., Liang H., J Bai Y. (1997). The Chemical Constituents from the Roots of Bupleurum chinense DC. *Journal of Chinese Pharmaceutical Sciences*.

[59] Liang H., Zhao Y. Y., Zhang R. Y. (1998). A new chromone glycoside from Bupleurum chinense. *Chinese Chemical Letters*.

[60] Liang H., Zhao Y., Cui Y., Liu Q. (2000). Flavonoids from the roots of Bupleurum chinense DC. *Journal of Beijing Medical University*.

[61] Tan L., Zhao Y. Y., Wang B. (1998). New isoflavonoside from Bupleurum scorzonerifolium. *Chinese Chemical Letters*.

[62] Chang W.-L., Chiu L.-W., Lai J.-H., Lin H.-C. (2003). Immunosuppressive flavones and lignans from Bupleurum scorzonerifolium. *Phytochemistry*.

[63] Kobayashi M., Tawara T., Tsuchida T., Mitsuhashi H. (1990). Studies on the constituents of Umbelliferae plants. XVIII. Minor constituents of Bupleuri Radix: Occurrence of saikogenins, polyhydroxysterols, a trihydroxy C18 fatty acid, a lignan and a new chromone. *Chemical and Pharmaceutical Bulletin*.

[64] Li Q. C., Liang H., Zhao Y. Y. (1997). The chemical constituents from the roots of bupleurum chinense. *Journal of Chinese Pharmaceutical Sciences*.

[65] Li X. Q., Song A. H., Li X W., Bi K. S. (2006). Analysis of the fatty acid from Bupleurum chinense DC in China by GC-MS and GC-FID. *Chemical & Pharmaceutical Bulletin*.

[66] Xie J. Y. (2012). Bupleurum chinense DC polysaccharides attenuates lipopolysaccharide-induced acute lung injury in mice. *Phytomedicine International Journal of Phytotherapy & Phytopharmacology*.

[67] Chun J., Tosun A., Kim Y. S. (2016). Anti-inflammatory effect of corymbocoumarin from Seseli gummiferum subsp. corymbosum through suppression of NF-*κ*B signaling pathway and induction of HO-1 expression in LPS-stimulated RAW 264.7 cells. *International Immunopharmacology*.

[68] Zhu J., Luo C., Wang P., He Q., Zhou J., Peng H. (2013). Saikosaponin A mediates the inflammatory response by inhibiting the MAPK and NF-*κ*B pathways in LPS-stimulated RAW 264.7 cells. *Experimental and Therapeutic Medicine*.

[69] Fu Y., Hu X., Cao Y., Zhang Z., Zhang N. (2015). Saikosaponin a inhibits lipopolysaccharide-oxidative stress and inflammation in Human umbilical vein endothelial cells via preventing TLR4 translocation into lipid rafts. *Free Radical Biology and Medicine*.

[70] Lee T. H., Chang J., Kim B. M. (2014). Saikosaponin C inhibits lipopolysaccharide-induced apoptosis by suppressing caspase-3 activation and subsequent degradation of focal adhesion kinase in human umbilical vein endothelial cells. *Biochemical and Biophysical Research Communications*.

[71] Zhao H., Li S., Zhang H. (2015). Saikosaponin a protects against experimental sepsis via inhibition of NOD2-mediated NF-*κ*B activation. *Experimental and Therapeutic Medicine*.

[72] Kodama Y., Xiaochuan L., Tsuchiya C., Ohizumi Y., Yoshida M., Nakahata N. (2003). Dual effect of saikogenin D: in vitro inhibition of prostaglandin E 2 production and elevation of intracellular free Ca^2+^ concentration in C6 rat glioma cells. *Planta Medica*.

[73] Li P., Kawachi H., Morioka T. (1997). Suppressive effects of Sairei-to on monoclonal antibody 1-22-3-induced glomerulonephritis: Analysis of effective components. *Pathology International*.

[74] Chen Y., Zhou X. L., Xue C. R. (2010). Therapeutic effects and mechanisms of chaihushugan decoction in experimental chronic pancreatitis rats. *Chinese Journal of Surgery of Integrated Traditional & Western Medicine*.

[75] Liu J., Zhao Z., Xue C. R. (2010). Effect of chaihu shugan pulvis on dysfunction of pancreatic exocrine secretion in patients with chronic pancreatitis. *Chinese Journal of Surgery of Integrated Traditional & Western Medicine*.

[76] Lenon G. B., Li C. G., Xue C. C., Thien F. C. K., Story D. F. (2008). Inhibition of inducible nitric oxide production and iNOS protein expression in lipopolysaccharide-stimulated rat aorta and Raw 264.7 macrophages by ethanol extract of a Chinese herbal medicine formula (RCM-101) for allergic rhinitis. *Journal of Ethnopharmacology*.

[77] Cheng Y.-L., Chang W.-L., Lee S.-C. (2003). Acetone extract of Bupleurum scorzonerifolium inhibits proliferation of A549 human lung cancer cells via inducing apoptosis and suppressing telomerase activity. *Life Sciences*.

[78] Chen Y.-L., Lin S.-Z., Chang W.-L., Cheng Y.-L., Harn H.-J. (2005). Requirement for ERK activation in acetone extract identified from Bupleurrum scorzonerifolium induced A549 tumor cell apoptosis and keratin 8 phosphorylation. *Life Sciences*.

[79] Cheng Y.-L., Lee S.-C., Lin S.-Z. (2005). Anti-proliferative activity of Bupleurum scrozonerifolium in A549 human lung cancer cells in vitro and in vivo. *Cancer Letters*.

[80] Hsu Y.-L., Kuo P.-L., Weng T.-C., Yen M.-H., Chiang L.-C., Lin C.-C. (2004). The antiproliferative activity of saponin-enriched fraction from Bupleurum kaoi is through Fas-dependent apoptotic pathway in human non-small cell lung cancer A549 cells. *Biological and Pharmaceutical Bulletin*.

[81] Chiang L.-C., Ng L. T., Liu L.-T., Shieh D.-E., Lin C.-C. (2003). Cytotoxicity and anti-hepatitis B virus activities of saikosaponins from *Bupleurum* species. *Planta Medica*.

[82] Hou H. L., He S. X., Zhu Z. F. (2011). “The role of saikosaponin d in regulating HIF-1*α*/COX-2 signal transduction pathway in human hepatocellular carcinoma cells. *Journal of Xian Jiaotong University*.

[83] Willimott S., Barker J., Jones L. A., Opara E. I. (2009). An in vitro based investigation of the cytotoxic effect of water extracts of the Chinese herbal remedy LD on cancer cells. *Chemistry Central Journal*.

[84] Kuroki T. (1995). Prospective study of chemoprevention of hepatocellular carcinoma with sho-saiko-to (TJ-9). *Cancer*.

[85] Zhao H. Y. (1995). Clinical observation of the treatment of primary liver cancer by using xiao-chai-hu decoction. *Journal of Traditional Chinese Medicine*.

[86] Haranaka K., Satomi N., Sakurai A., Haranaka R., Okada N., Kobayashi M. (1985). Antitumor activities and tumor necrosis factor producibility of traditional Chinese medicines and crude drugs. *Cancer Immunology Immunotherapy*.

[87] Chang J.-S., Wang K.-C., Liu H.-W., Chen M.-C., Chiang L.-C., Lin C.-C. (2007). Sho-saiko-to (Xiao-Chai-Hu-Tang) and crude saikosaponins inhibit Hepatitis B virus in a stable HBV-producing cell line. *American Journal of Chinese Medicine*.

[88] Yin F., Pan R., Chen R., Hu L. (2008). Saikosaponins from Bupleurum chinense and inhibition of HBV DNA replication activity. *Natural Product Communications*.

[89] Cheng P. W., Ng L. T., Chiang L. C., Lin C. C. (2006). Antiviral effects of saikosaponins on human coronavirus 229E in vitro. *Clinical & Experimental Pharmacology & Physiology*.

[90] Jin G. T., Bo L., Wang S. R. (2013). Experimental study on material basis, efficacy and mechanism of antipyretic effect of Bupleuri Radix. *Journal of Chengdu University of Traditional Chinese Medicine*.

[91] Cao S.-L., Chen E., Zhang Q.-Z., Jiang X.-G. (2007). A novel nasal delivery system of a Chinese traditional medicine, Radix bupleuri, based on the concept of ion-activated in situ gel. *Archives of Pharmacal Research*.

[92] Xie Y., Lu W., Cao S., Jiang X., Yin M., Tang W. (2006). Preparation of bupleurum nasal spray and evaluation on its safety and efficacy. *Chemical and Pharmaceutical Bulletin*.

[93] Li Y., Xu C., Zhang Q., Liu J. Y., Tan R. X. (2005). In vitro anti-Helicobacter pylori action of 30 Chinese herbal medicines used to treat ulcer diseases. *Journal of Ethnopharmacology*.

[94] Kumazawa Y., Kawakita T., Takimoto H., Nomoto K. (1990). Protective effect of saikosaponin a, saikosaponin d and saikogenin D against Pseudomonas aeruginosa infection in mice. *International Journal of Immunopharmacology*.

[95] Du X. M., Xie H., Kang Y. (2006). The Pharmacological activity of chinese “chaihu”-the root of bupleurum species. *Sci Eval Clin Appl*.

[96] Li Z.-Y., Sun H.-M., Xing J., Qin X.-M., Du G.-H. (2015). Chemical and biological comparison of raw and vinegar-baked Radix Bupleuri. *Journal of Ethnopharmacology*.

[97] Abe H., Sakaguchi M., Yamada M., Arichi S., Odashima S. (1980). Pharmacological actions of saikosaponins isolated from Bupleurum falcatum. I. Effects of saikosaponings on liver function. *Planta Medica*.

[98] Matsuda H., Murakami T., Ninomiya K., Inadzuki M., Yoshikawa M. (1997). New hepatoprotective saponins, bupleurosides III, VI, IX, and XIII, from Chinese Bupleuri Radix: Structure-requirements for the cytoprotective activity in primary cultured rat hepatocytes. *Bioorganic & Medicinal Chemistry Letters*.

[99] Han X. H., Gai X. D., Xue Y. J., Chen M. (2006). Effects of the extracts from Bupleurum Chinese DC on intracelluar free calcium concentration and vincristine accumulation in human hepatoma BEL-7402 cells. *Tumor*.

[100] Abe H., Sakaguchi M., Odashima S., Arichi S. (1982). Protective effect of saikosaponin-d isolated from Bupleurum falcatum L. on CCl4-induced liver injury in the rat. *Naunyn-Schmiedeberg's Archives of Pharmacology*.

[101] Fan J., Li X., Li P. (2007). Saikosaponin-d attenuates the development of liver fibrosis by preventing hepatocyte injury. *Biochemistry & Cell Biology*.

[102] Yamakage M., Hattori J.-I., Satoh J.-I., Namiki A. (2006). Effects of the Chinese herbal medicines Bupleuri radix, Ginseng radix, and Zingiberis rhizoma on lymphatic vessel activity in rats. *American Journal of Chinese Medicine*.

[103] Kumazawa Y., Takimoto H., Nishimura C., Kawakita T., Nomoto K. (1989). Activation of murine peritoneal macrophages by saikosaponin a, saikosaponin d and saikogenin d. *International Journal of Immunopharmacology*.

[104] Kato M., Pu M.-Y., Isobe K.-I., Hattori T., Yanagita N., Nakashima I. (1995). Cell type-oriented differential modulatory actions of saikosaponin-d on growth responses and DNA fragmentation of lymphocytes triggered by receptor-mediated and receptor-bypassed pathways. *Immunopharmacology*.

[105] Kato M., Pu M.-Y., Isobe K.-I. (1994). Characterization of the immunoregulatory action of saikosaponin-d. *Cellular Immunology*.

[106] Wong V. K. W., Zhou H., Cheung S. S. F., Li T., Liu L. (2009). Mechanistic study of saikosaponin-d (Ssd) on suppression of murine T lymphocyte activation. *Journal of Cellular Biochemistry*.

[107] Yi H., Long B., Ye X., Zhang L., Liu X., Zhang C. (2014). Autophagy: A potential target for thyroid cancer therapy (Review). *Molecular and Clinical Oncology*.

[108] Mizushima N. (2007). Autophagy: process and function. *Genes & Development*.

[109] Rubinsztein D. C., Codogno P., Levine B. (2012). Autophagy modulation as a potential therapeutic target for diverse diseases. *Nature Reviews Drug Discovery*.

[110] Yang Z. J., Chee C. E., Huang S., Sinicrope F. A. (2011). The role of autophagy in cancer: therapeutic implications. *Molecular Cancer Therapeutics*.

[111] Eskelinen E.-L. (2011). The dual role of autophagy in cancer. *Current Opinion in Pharmacology*.

[112] Yamaguchi O., Otsu K. (2012). Role of autophagy in aging. *Journal of Cardiovascular Pharmacology*.

[113] Law B. Y., Mo J. F., Wong V. K. (2014). Autophagic effects of *Chaihu* (dried roots of *Bupleurum Chinense DC* or *Bupleurum scorzoneraefolium WILD*). *Chinese Medicine*.

[114] Cheng Y., Huang Y., Tian Y., Xu L., Liu G. Q., Zahng Z. J. (2013). Assessment of the effects of Radix Bupleuri and vinegar-baked Radix Bupleuri on cytochrome 450 activity by a six-drug cocktail approach. *Chinese Journal of Natural Medicines*.

[115] Chen X., Yu T., Chen Z., Zhao R., Mao S. (2014). Effect of saikosaponins and extracts of vinegar-baked Bupleuri Radix on the activity of *β*-glucuronidase. *Xenobiotica*.

[116] Ye M., Bi Y.-F., Ding L., Zhu W.-W., Gao W. (2016). Saikosaponin a functions as anti-epileptic effect in pentylenetetrazol induced rats through inhibiting mTOR signaling pathway. *Biomedicine and Pharmacotherapy*.

[117] He D., Wang H., Xu L. (2016). Saikosaponin-a Attenuates Oxidized LDL Uptake and Prompts Cholesterol Efflux in THP-1 Cells. *Journal of Cardiovascular Pharmacology*.

[118] Shyu K.-G., Tsai S.-C., Wang B.-W., Liu Y.-C., Lee C.-C. (2004). Saikosaponin C induces endothelial cells growth, migration and capillary tube formation. *Life Sciences*.

[119] Lee T. H., Park S. H., You M.-H., Lim J.-H., Min S.-H., Kim B. M. (2016). A potential therapeutic effect of saikosaponin C as a novel dual-target anti-Alzheimer agent. *Journal of Neurochemistry*.

[120] Zhu S., Ling F., Zhang Q. (2014). Anthelmintic activity of saikosaponins a and d from radix bupleuri against Dactylogyrus spp. infecting goldfish. *Diseases of Aquatic Organisms*.

[121] Cui S. C., Cao Q. Z., Su C. X. (1999). Analysis of 50 cases of drug-induced liver disease. *Journal of Capital Medicine University*.

[122] Teo D. C. H., Ng P. S. L., Tan S. H. (2016). Drug-induced liver injury associated with Complementary and Alternative Medicine: A review of adverse event reports in an Asian community from 2009 to 2014. *BMC Complementary and Alternative Medicine*.

[123] Itoh S., Marutani K., Nishijima T., Matsuo S., Itabashi M. (1995). Liver injuries induced by herbal medicine, Syo-saiko-to (xiao-chai-hu-tang). *Digestive Diseases and Sciences*.

[124] Lee C.-H., Wang J.-D., Chen P.-C. (2011). Risk of Liver Injury Associated with Chinese Herbal Products Containing Radix bupleuri in 639, 779 Patients with Hepatitis B Virus Infection. *PLoS ONE*.

[125] Sun R., Wang L., Yang Q., Huang W., Lv L. L. (2010). Acute toxicity of volatile oil from Bupleurum chinense in rats and mice. *Chinese Journal of Experimental Traditional Medical Formulae*.

[126] Huang W., Sun R., Zhang Z. (2010). “Dose-time-toxicity” relationship study on hepatotoxicity caused by multiple dose of total Bupleurum saponin crude extracts to rats. *China Journal of Chinese Materia Medica*.

[127] Xu L., Song R., Tian J.-X., Tian Y., Liu G.-Q., Zhang Z.-J. (2012). Analysis of saikosaponins in rat plasma by anionic adducts-based liquid chromatography tandem mass spectrometry method. *Biomedical Chromatography*.

[128] Lin L., Ni B., Lin H. (2015). Traditional usages, botany, phytochemistry, pharmacology and toxicology of *Polygonum multiflorum* Thunb.: a review. *Journal of Ethnopharmacology*.

